# The Phe-Phe Motif for Peptide Self-Assembly in Nanomedicine

**DOI:** 10.3390/molecules201119658

**Published:** 2015-11-03

**Authors:** Silvia Marchesan, Attilio V. Vargiu, Katie E. Styan

**Affiliations:** 1 Chemical and Pharmaceutical Sciences Department, University of Trieste, Via L. Giorgieri 1, Trieste 34127, Italy; 2 Department of Physics, University of Cagliari, Cittadella Universitaria S.P. Monserrato-Sestu Km. 0.700, Monserrato 09042, Italy; vargiu@dsf.unica.it; 3 CSIRO Manufacturing, Bayview Ave Clayton, VIC 3168, Australia; katie.styan@csiro.au

**Keywords:** diphenylalanine, self-assembly, hydrogels, peptides, nanostructures

## Abstract

Since its discovery, the Phe-Phe motif has gained in popularity as a minimalist building block to drive the self-assembly of short peptides and their analogues into nanostructures and hydrogels. Molecules based on the Phe-Phe motif have found a range of applications in nanomedicine, from drug delivery and biomaterials to new therapeutic paradigms. Here we discuss the various production methods for this class of compounds, and the characterization, nanomorphologies, and application of their self-assembled nanostructures. We include the most recent findings on their remarkable properties, which hold substantial promise for the creation of the next generation nanomedicines.

## 1. Introduction

Nanotechnologies are revolutionizing the medicinal field as they promise innovative solutions to unsolved therapeutic and diagnostic challenges [1]. The unique physico-chemical properties of nanomaterials allow for unprecedented performance, especially in the field of early diagnosis and targeted drug delivery [2]. It is not just a quantitative leap in, say, the efficiency of disease identification and treatment that is on the horizon, but also a qualitative leap that can be realized by nanomaterials through innovative therapeutic paradigms, as we will discuss below.

Amongst the wide variety of molecules that are active players in this nanotechnology field, peptides have gained widespread popularity for a number of reasons [3]. First, they can deliver powerful and selective biological messages to cells, thus would seem ideal candidates for biological applications, assuming any immunological response can be managed. Second, they can be prepared by a number of means, including solid- and liquid-phase chemistry, biocatalysis, metabolic engineering, and recombinant expression biotechnologies. In other words, they are easily accessible not only to chemists, but also to biologists, biochemists, and materials scientists who may not be capable of overcoming the synthetic challenges posed by other non-peptidic small molecules. Third, peptides have been extensively chemically and biologically characterized, with the peptidic final product having a well-defined chemical composition. Peptide chemistry is well-established, highly reproducible, and it allows easy incorporation of non-peptidic moieties [4]. This is contrary to purified proteins, which typically exist as a mixture of components (e.g., proteins with different post-translational modifications and various conjugated glycans). Thus there is ample grounds to safely predict a peptides’ molecular behavior in a variety of experimental contexts and so their use is generally well-accepted by a number of communities ranging from scientists, to the public, and to regulatory bodies. Fourth, they are readily biodegradable and are not expected to present any threat to the environment, which is an aspect that is rightly being taken into serious consideration nowadays. In fact, biodegradability is often considered a short-coming for traditional application of peptides, and methods such as use of synthetic analogues and encapsulation as protection from enzymatic hydrolysis, have been employed to overcome this. Finally, the ability of peptides (and proteins) to spontaneously fold into complex and flexible 3D structures, which could be used to mediate catalysis or other functional or structural tasks, is attractive. This versatility, alongside the peptides’ functional groups and physico-chemical properties, offers an ideal playground for the imaginative scientist to employ them in the design of new nanotechnological tools, bulk nanomaterials, or adaptive nanostructures.

Within the peptide field, self-assembling short peptides have stolen the scene from the more cumbersome analogues such as proteins and polypeptides [5]. This is easily explained by the numerous advantages of the former relative to the latter, such as ease and relative low cost of preparation, greater stability, simpler characterization, and, generally easier handling for the novice in the field. From a chemical point of view, if we look at the minimalist structures of low molecular weight self-assembling peptides, we can easily recognize a powerful yet simple design approach that has been successfully used by several research groups. Amongst the various non-covalent interactions that can be employed for self-assembly, π-π stacking plays a special role since it facilitates the persistent supramolecular association of compounds of extreme chemical simplicity to provide self-assembled structures of high stability. Remarkably, it seems sufficient to bind any appropriate aromatic unit to a range of diverse di- or tri-peptides, or even to single amino acids, to generate a self-assembling motif suitable for aqueous environments. The most often used aromatic structure is probably the fluorenylmethyloxycarbonyl group (*i.e*., Fmoc), thanks to its extensive use in peptide synthesis, and thus its ready commercial availability pre-linked to any amino acid of choice. Other popular options amongst chemists are naphthalene, anthracene, and benzyloxy derivatives [6,7].

The powerful forces of π-π stacking interactions, such as presented in the relatively simple diphenylalanine short peptide motif, may be sufficient to enable access to a wide variety of nanostructures [8]. This motif lies at the core of the Amyloid β peptide self-assembling sequence, and was identified through a systematic reductionist approach aimed at searching for the smallest recognition module for self-assembly [8]. Remarkably, even the amino acid phenylalanine was shown to form self-assembled fibers [9]. Indeed, the so-called “Phe-Phe motif” is a surprisingly simple chemical structure that has only recently revealed its full potential in self-assembly, as shown by the huge number of scientific reports spanning from the chemical and materials fields, to medicine and biology applications [10]. To celebrate its surprisingly versatile supramolecular behavior, which has been finely controlled through elegant studies for innovative applications in nanomedicine, we chose the Phe-Phe motif as the topic of this mini-review. Here, we discuss the preparation, characterization, and use of the class of molecules bearing the Phe-Phe motif, with a special emphasis on the rapid progress they are realizing in nanomedicine.

## 2. Preparation of Compounds Bearing the Phe-Phe Motif

One of the advantages of short peptides is their ease of preparation by a wide number of techniques, although thanks to the wide availability of commercial sources it is not uncommon to find studies using purchased compounds [11,12,13,14]. Possible methods for their preparation include solid- [15] and liquid-phase [16,17] chemical synthesis, enzymatic catalysis [18,19], and even biotechnology methods that make use of genetically modified organisms [20]. We provide here a brief overview of the different methods, including their advantages and disadvantages, as well as their often unrecognized potential to give access to different classes of peptide molecules. We will not discuss purification, except to briefly state in most cases this is achieved by HPLC, although other chromatographic, or even phase-separation methods (e.g., precipitation, crystallization), are often employed. Readers interested in this aspect are referred to extensive reviews on the topic [21,22].

From a synthetic chemistry point of view, short peptides can be easily prepared by solid-phase peptide synthesis (SPPS) [15], of which the Fmoc chemistry is so well-known and established that it won’t be described in detail here. Today chemists can take advantage of the large commercial availability of resins (e.g., Wang, chlorotrityl and Rink-amide are popular options), coupling agents (e.g., phosphonium or aminium/uronium-imonium derivatives, carbodiimides, *etc.*), suppressors of racemization (e.g., benzotriazole- or oxyma-derivatives), and dedicated solvents (e.g., amine-free dimethylformamide and high quality dry dichloromethane). Detailed synthetic protocols and troubleshooting guides can be easily accessed by the non-expert in the field to ensure a successful synthesis. From a technical point of view, the simplicity of SPPS makes it a viable synthetic option even for the non-chemist. Modern tools, including fully automated peptide synthesizers that can provide peptides in a matter of hours make this especially true, although this of course comes at an economic cost that may make it an unviable option for many.

An alternative for the skilled organic chemist is liquid-phase synthesis, which can be employed to prepare short peptides or their derivatives, with the number of amino acids being limited by the expertise of the chemist and the inherent synthetic challenges posed by each particular peptide sequence. This approach is cheaper than SPPS and more readily scalable, but if one decides to use it for relatively complex molecules, the subsequent compound purification can be onerous. This labor intensive step is probably the reason liquid-phase synthesis is mainly employed to prepare dipeptides and their derivatives [17], and occasionally tripeptides [16], rather than longer sequences. In addition, this approach generally requires far more attention than SPPS to be successful, to the point that it may pose a serious challenge to the inexperienced. Nevertheless, if the molecule to prepare is fairly simple, this method is probably the best choice to rapidly prepare large amounts of compounds at a very reasonable cost.

Enzymatic catalysis is another method that is gaining in popularity in recent years. This is probably the result of a combination of factors, including the increasing availability of industrial enzymes that are both low cost and good catalysts for a wide range of reactions, including unnatural peptides and even other chemical classes of compounds, thus offering affordable reproducibility and substrate promiscuity. For instance, ligases can synthesize oligopeptides, albeit in an ATP-dependent manner [23]. The toolbox of enzymes that can be used for this purpose has been broadened to include their use in “reverse”, *i.e*., the application of proteases [18] and, more generally, hydrolases [19] (e.g., lipases, esterases, *etc.*) to link together molecules in a newly formed covalent bond (*i.e*., typically a peptide bond for Phe-Phe derivatives). This approach can be applied also to unnatural amino acids [19] and their derivatives, such as those *N*-terminally protected, thanks to enzymatic tolerance towards different substrates [24]. The increasing importance placed on environmental impact makes this approach preferable over SPPS or liquid-phase chemistries mentioned above, since it avoids the creation of hazardous waste and the use of toxic solvents. Indeed, most reactions can be conducted in water or in relatively environmentally benign non-halogenated solvents such as alcohols. Additionally, reactions often occur at room temperature or require only mild heating, thus making the process attractive from an energetic point of view. Overall, this approach is probably the most attractive both for ecological reasons and for social acceptance.

Another less known and used avenue utilizes biotechnologies, or recombinant techniques, that are typically used to produce higher-molecular-weight compounds that are not easily accessible by other means (e.g., full-length proteins or large polypeptides). That said, recombinant expression in bacteria of short peptides has been reported [20]. Creation of new metabolic pathways is also possible to produce single amino acids of natural or “unnatural” origin (e.g., with d-stereoconfiguration) [25]. Ease of large-scale preparation and reproducibility make these routes viable options for industry, however, optimization of the experimental conditions is not always straightforward nor easy to predict. Moreover, handling of genetically modified organisms requires a set of biosafety measures, training, and dedicated instrumentation that is often not accessible to the chemists wanting to study self-assembling compounds.

Finally, although not a currently used source, it would be extremely interesting to develop methods to extract and isolate this class of molecules from natural sources. One may think naturally that only l-peptides can be accessed by this route, however we should remember that d-peptides can also be found in natural sources [26], including the marine environment [27]. Identification of low-cost sources of self-assembling building blocks, such as algae or jellyfish, would be highly attractive for industrial use.

## 3. Characterization of Compounds Bearing the Phe-Phe Motif

If we consider the peptidic nature of the Phe-Phe motif, we immediately identify a number of traditional techniques that can be used to characterize these compounds. Mass spectroscopy, HPLC, NMR, FT-IR, and UV-Vis, are all well-established characterization methods that can provide useful data on both the molecule and its supramolecular arrangements. Circular dichroism and polarimetry are perhaps more familiar to the biochemist, and they provide key information on the chiral nature of these compounds, and again, of their supramolecular assemblies. The application of these techniques to self-assembled hydrogels and nanostructures is not always trivial from a purely technical point of view, thus works that publish step-by-step videos of experimental procedures can provide useful guidance [28].

From a supramolecular perspective, several nanoscale microscopy techniques, such as AFM [29], TEM [30], STM [31], and SEM [32], can provide compelling images that reveal fine nanostructure detail. Nowadays even light microscopy can be a useful tool in the field of nanomaterials thanks to the realization of super-resolution instrumentation to unveil features in the relatively remarkably high resolution of a few tens of nanometers [33]. Self-assembled peptides of amyloid nature can be visualized by using specific stains such as Thioflavin T [34] or Congo Red [35].

Thermal analyses such as DSC and TGA provide a useful means to probe these nanomaterials and the stability of their assembled nanostructures. Use of these techniques is uncommon for self-assembled peptides though, with only a few reports detailing such characterization [36,37,38,39].

X-Ray diffraction and SAXS are other methods that can provide very valuable information on the self-assemblies [38], but they are unfortunately not always a viable option. XRD often requires high-quality crystals, or at least highly ordered assemblies, for the detection of signals from low molecular weight peptides. That said, XRD has been successfully applied to unravel the ordered assembly of hydrophobic dipeptides, such as Phe-Phe into nanotubes [40]. SAXS may be an easier alternative from a technical point of view, but even in this case the generation of a detectable signal from short peptides may require use of more intense synchrotron light radiation [41].

NMR can provide useful information about the self-assembly process, if the difficulties often associated with the gelation of these systems are overcome. For example, ^1^H-NMR diffusion-ordered spectroscopy (DOSY) was successfully applied to investigate the kinetics of gelation of Fmoc-Phe-Phe, showing that gelation is associated with a first order phase transition, nucleation, and growth of elongated crystals [11]. Additionally, a DOSY technique was recently developed to detect the presence of intermediate oligomers of diphenylalanine in solution during its self-assembly into nanotubes, through the rapid characterization of translational diffusion of molecules [42].

Molecular modelling is another technique that can reveal key features of supramolecular assemblies, and indeed it has been increasingly applied over the past decade to decipher how small changes in the primary structure can lead to significantly different nanostructures with diverse functions [43,44,45]. Computational modeling was successfully employed to unveil the most diverse aspects of supramolecular self-assembly as recently reviewed [44]. In particular, molecular dynamics in different flavors has been applied by several groups to investigate some properties of di- and tri-phenylalanine peptides, such as: peculiar structural motifs stabilizing their supramolecular structures; the nature of the main forces driving self-assembly; the influence of the solvent and of electric field on the kinetics and mechanism of self-assembly; the overall shape of the supramolecular structures obtained at different conditions [46,47,48,49,50,51]. Furthermore, coarse-grained molecular dynamics was recently employed to investigate the self-assembly propensities of all possible l-amino acid combinations forming di- and tri-peptides [52,53]. Yet the influence of chirality on self-assembly and supramolecular order was poorly investigated *in silico*. According to our knowledge, only two computational studies (both based on experimental data) have been published to date on the topic, offering interesting models capable of revealing phenylalanine zippers [54] or other features such as the formation of “dry” regions in highly ordered heterochiral peptide stacks [36].

## 4. Nanomorphologies Formed by the Phe-Phe Motif

**Figure 1:**
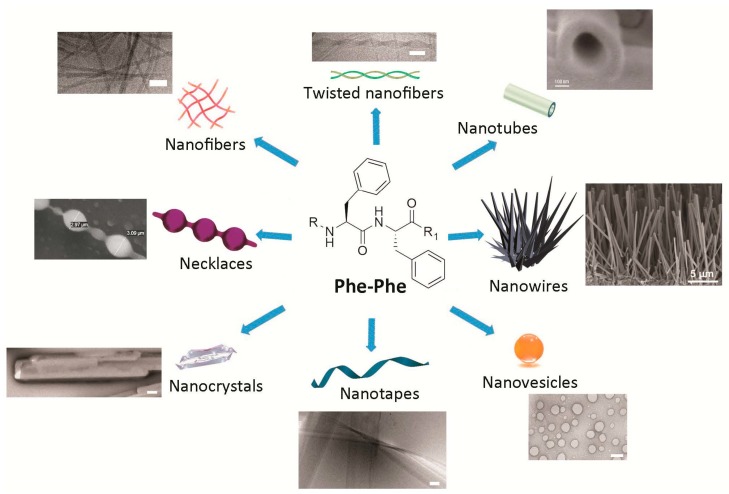
Simple compounds bearing the Phe-Phe motif self-assemble into diverse nanomorphologies. Scale bars = 100 nm, unless otherwise stated on the image. Adapted with permission from: refs. [13,55] © 2012 American Chemical Society; refs. [36,54,56] with kind permission of the Royal Society of Chemistry; ref. [39] with permission from Wiley © 2008 WILEY-VCH Verlag GmbH & Co. KGaA; and with permission from ref. [57] © 2006 Nature Publishing Group.

The Phe-Phe motif is an extremely versatile self-assembling building block. Interestingly, subtle changes introduced to the chemical structure of the Phe-Phe derivative are sufficient to obtain different nanomorphologies (Figure 1). Alternatively, assembly of any one compound can be driven towards distinct nanoassemblies by varying the experimental conditions. In either case, there is yet no general over-arching principle for the *ex novo* design of specific nanostructures, although the understanding of these systems, and the ability to predict their supramolecular behavior, is rapidly progressing as more reports are generated on the topic.

Nanotubes as long as hundreds of microns were first reported for Phe-Phe upon the dilution of a highly concentrated solution of 100 mg/mL of the dipeptide in hexafluoroisopropanol, in water. This elegant study demonstrated the hollow nature of the tubes by using them as templates for the reduction of silver salts to form inorganic nanowires within the nanotubes. The nanowires were easily visible upon protease-mediated degradation of the peptide [8]. As an aside, amidation of the carboxyl terminal of Phe-Phe does not hamper formation of the nanostructures [12].

The same experimental protocol as described above can result in spherical vesicles when applied to the more rigid diphenylglycine or to a thiol containing derivative (*i.e*., Cys-Phe-Phe tripeptide or Phe-Phe reacted with Traut’s reagent). Remarkably, the nanovesicles are stable to a wide range of pH values, including both alkaline (*i.e*., 1 M sodium hydroxide) or acidic (*i.e*., 10% trifluoroacetic acid) conditions, thus giving scope for their adoption in biomedical applications [13]. Alternatively, amidated Phe-Phe dipeptide assemblies can undergo structural transition from nanotubes, through a necklace-like intermediate state, to individually dispersed spherical vesicles upon dilution at physiological pH, thus showing that concentration plays a crucial role in the process [12]. The Phe-Phe dipeptide can form nanovesicles too, instead of nanotubes, upon further dilution in water [58]. Spherical assemblies are also obtained upon introduction of two azide groups, each one in *para* on the aromatic ring of the phenylalanine of the dipeptide, where the azides could serve as photo-crosslinking moieties to fine-tune viscoelastic properties through the use of light. By contrast, the presence of only one azido-Phe does not lead to such nanomorphologies [59].

A network of nanofibrils giving rise to a hydrogel is obtained if the dipeptide Phe-Phe is substituted at the *N*-terminal with aromatic synthetic moieties such as fluorenylmethyloxycarbonyl (*i.e*., Fmoc), or naphthalene units [24]. Alternatively, addition of leucine or isoleucine as a third, hydrophobic amino acid in a tripeptide with d-l-l stereoconfiguration (*i.e*., ^d^Leu-Phe-Phe [54] or ^d^Phe-Phe-Ile [60]) yields a gelling heterochiral tripeptide with self-assembled fibrils rapidly bundling into thicker fibers.

Nanotapes as wide as tens of nanometers and as long as a few hundred microns are formed by heterochiral stereoisomers of the sequence Val-Phe-Phe upon pH change form alkaline (*i.e*., pH >11.5) to neutral (*i.e*., pH 7.4). Depending on the position of the d-amino acid(s) along the tripeptide, supramolecular assemblies are formed with higher or lower order, giving rise to hydrogels with different rheological and thermal behavior [36].

Fibers twisted in pairs with a recurrent helical period are obtained with the heterochiral ^d^Phe-Phe-Val or its enantiomer Phe-^d^Phe-^d^Val, when the same pH trigger as above is used to obtain the self-assembling zwitterions from a solution of the anionic peptide in alkaline buffer (*i.e*., pH ≥ 11.5) to allow for peptide dissolution at concentrations as high as 30 mM). The resulting matrix holds high amounts of water in a hydrogel at physiological pH [56].

Combination of different self-assembling peptides to achieve more complex nanomorphologies is also possible. For instance, mixtures of nanosphere-forming Boc-Phe-Phe and nanotube-forming Phe-Phe allow for the formation of nanosized “necklaces”, where chains of beads are connected by elongated structures [55].

Switching between peptide nanomorphologies can be achieved upon appropriate chemical design, for instance through insertion of light-responsive units such as azobenzenes that, through photoinduced *cis-trans* conformational change, permit the reversible switch from vesicles to nanofibers [7].

Solid-phase growth of Phe-Phe self-assembled structures is another method that can be applied to achieve vertically aligned nanowires. The technique can be combined with soft-lithography to obtain micropatterned nanotopographies of remarkable thermal stability up to 200 °C [39]. Alignment of Phe-Phe nanotubes can be achieved with other approaches too, leading to vertical forests through solvent evaporation, or to horizontally aligned nanotubes through use of a ferrofluid and application of a magnetic field [57]. Such fine control over self-assembly could find innovative application in the field of flexible electroactive materials and sensors, for example.

## 5. Use for Drug-Delivery

An interesting avenue for drug delivery is the self-assembly of Phe-Phe derivatives and analogues into durable nanostructures that can enter cells. For example, it was recently shown that fluorinated β-peptides containing two aryl units can form nanotubes that are stable to protease degradation and heating up to 120 °C and can locate in the perinuclear region of cells. Cytotoxicity assays were performed on cultured primary human smooth muscle cells, which are derived from human blood vessels, and thus offer a good model to represent the most abundant cell type directly exposed to nanotherapeutics present in the blood system. After 48 h, no cytotoxic effect was noted for peptide concentrations ranging from 1 to 100 µM, whilst at 200 µM cell viability was significantly reduced to approximately 80% relative to the control [61].

Binding of a self-assembling Phe-Phe peptide and a small molecule(s), such as imaging agents or drugs, is an approach that can be used to improve the delivery of hydrophobic or unstable agents. The antitumor drug hydroxycamphothecin has been delivered to cells through the internalization of the self-assembled nanofibers to which it was bound, with a sustained drug release over a one-week period. Use of d-amino acids in the sequence provided excellent protease stability, allowing for prolonged therapeutic effect as observed *in vivo* over several weeks, with reduction of a tumor mass in a rat model accompanied by neither significant weight loss of the animal nor histopathological abnormalities or lesions in liver and spleen tissues [62].

**Figure 2:**
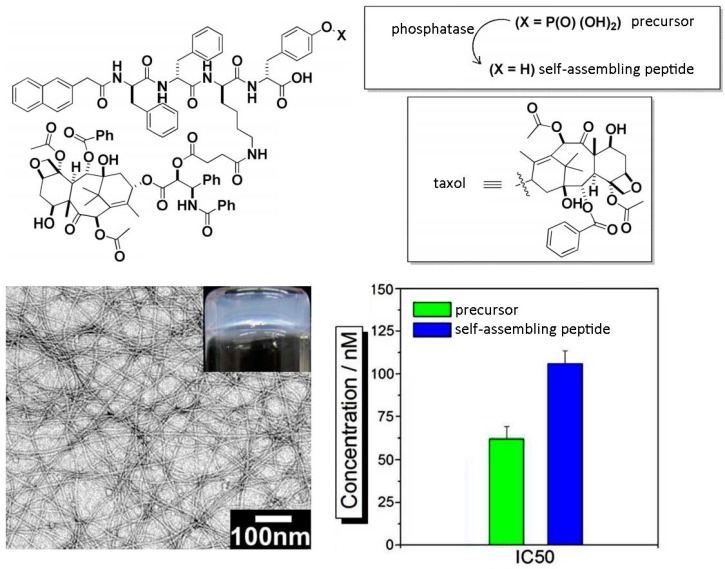
Chemical structure of a self-assembling peptide derivative covalently linked to taxol and formed upon phosphatase conversion of a phosphate precursor (**top**); upon self-assembly, a nanostructured hydrogel was formed and the IC_50_ calculated (**bottom**). Adapted with permission from ref. [63] © 2013 American Chemical Society.

A similar approach has been applied for the controlled release of the anticancer drug taxol, and of a fluorophore used as imaging agent *in vivo*, upon covalent binding of the small molecules to a self-assembling peptide motif (Figure 2). In particular, use of a phosphate precursor allows for the formation of the self-assembling derivative upon phosphatase-mediated enzymatic hydrolysis; preservation of taxol activity was confirmed through MTT assays on HeLa cells [63]. Co-delivery of dexamethasone and either taxol or 10-hydroxycaptothecin, two complementary drugs for cancer treatment, was achieved through the hydrolysis of ester linkages that covalently held the drugs attached onto a self-assembling Phe-Phe peptide. In this case, the resulting hydrogels displayed different stability over time at 37 °C, depending on their chemical structure (*i.e*., 48 h for the taxol-derivative, and over two weeks for the other). However, the use of mixtures containing both of the self-assembling components, or just one with the addition of albumin protein, was a useful approach to overall extend hydrogels stability over weeks. Such formulations proved effective for sustained drug delivery *in vitro*, while no peptide component was detected in the release medium, thus confirming the stability of the supramolecular assemblies. In addition, *in vitro* assays revealed no cytotoxic effect arising from the self-assembling peptide for concentrations up to 100 µM over 48 h [64]. Other useful applications include the development of hydrogels as vehicles for non-steroidal anti-inflammatory drugs (NSAIDs) for local use. In this case, unexpected advantages such as improved COX-2 enzyme selectivity, and correspondingly reduced side-effects, have been observed for the peptide-drug conjugate. In addition, no cytotoxic effect was observed over 72 h on HeLa cells treated with hydrogelator concentrations as high as over 300 µM, or even 500 µM [65].

Physical entrapment of small molecules in hydrophobic Phe-Phe-derived gel matrices is possible too, as shown for the controlled release of SPECT tracers *in vivo* [66]. Similarly, drugs can be loaded onto nanoparticles for slow release, and these can be entrapped in a hydrogel composed of Fmoc-Phe-Phe-Phe; use of homochiral or heterochiral Fmoc-tripeptide stereoisomers was not accompanied by cytotoxic effects on cells treated for 24 h [19].

**Figure 3:**
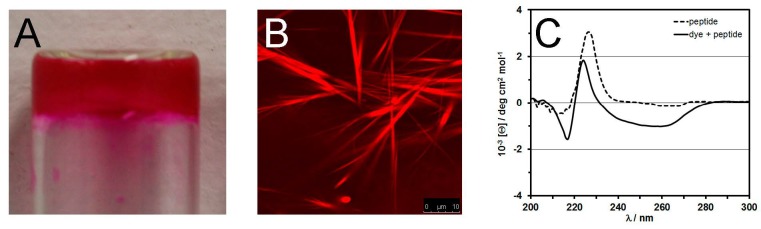
Rhodamine B actively participates in the assembly process of a tripeptide to yield a self-supportive hydrogel (**A**). Confocal fluorescence microscopy highlights the presence of the dye in the supramolecular structures (**B**); resulting in different circular dichroism spectra relative to the peptide alone (**C**) [68].

A more interesting alternative is the non-covalent binding of drugs [67] or imaging molecules [68] through their active participation in the self-assembly process of Phe-Phe peptides (Figure 3), allowing for different release kinetics upon disassembly of the nanostructured hydrogels. Also in this case no cytotoxic effect was observed either on fibroblast cultured on the hydrogel over 72 h, or in hemolysis assays performed with the release medium [67]. Spectroscopic evidence pointed to aromatic interactions between the peptide and the cargo, although electrostatic interactions may also play a role. Indeed, the latter were successfully employed for the binding of negatively-charged oligonucleotides that could be delivered inside cells, showing that there is wide scope for the delivery of (macro)molecular cargo [12].

## 6. Biomaterials Formed by Self-Assembly of Phe-Phe Derivatives

Diphenylalanine by itself does not form hydrogels, however, these can be obtained from self-assembling derivatives that, typically, employ synthetic aromatic units at the *N*-terminal [69], or from longer peptide sequences [70]. Although the former approach yields self-assembling motifs from simple amino acids and dipeptides, making it convenient from an economical point of view, there are concerns over cytotoxicity as reduced cell viability attributed to the *N*-terminal group has been reported for cells grown on hydrogels of this kind [71]. Therefore, there is a lot of interest in identifying self-organizing short peptides that do not require such synthetic capping groups [53]. Three is the minimum number of amino acids reported to have the capacity to form hydrogels at physiological pH without the use of organic solvents or synthetic capping groups; examples are the hydrophobic sequences Val-Phe-Phe, Leu-Phe-Phe, and Phe-Phe-Val [15,54]. Interestingly, it is only the heterochiral sequences (*i.e*., bearing both d- and l-amino acids at selected positions along the tripeptide) that form nanostructured soft materials under such conditions, while their homochiral analogues do not [36,56]. It has been hypothesized that the heterochiral stereoisomers can rapidly adopt the appropriate conformation to interlock into phenylalanine zippers, while the homochiral tripeptides suffer from steric clashes that interfere with their ordered stacking [54].

The self-assembly process *per se* has a large influence on the resultant viscoelastic properties of the ultimately formed self-assembled material [72]. Indeed, while there is a lot of interest in the development of simple building blocks for soft biomaterials, such as tripeptides, the prediction of their supramolecular behavior is still very challenging, and a recent survey of all 20^3^ = 8000 natural amino acid combinations identified only four new hits of hydrogelling tripeptides [53]. Di- and tri-peptides have obvious advantages in terms of their simplicity, cost, and scalability of preparation relative to their longer or more complex derivatives [5]. Additionally, hydrogels formed through the self-organization of small molecules offer an alternative to natural proteins as extracellular matrix (ECM) biomaterial scaffolds, as they allow for defined chemical composition and it is thought that the nanofibril morphology and physicochemical properties mimic that of the ECM.

3D cell culture is another potential biomedical application. Cell infiltration and proliferation has been shown for Fmoc-Phe-Phe hydrogels [73]. Incorporation of more than one self-assembling building block, for example Fmoc-Phe-Phe and Fmoc-Arg-Gly-Asp, to include both a self-assembly driver and a biological function such as promotion of cell adhesion, opens the way to more sophisticated biomaterials [74]. High cell viability and proliferation was also shown for heterochiral Phe-Phe tripeptide hydrogels, including those with d-supramolecular chirality, thus raising interesting questions about cell interaction with biomaterial chirality [36,56,67].

**Figure 4:**
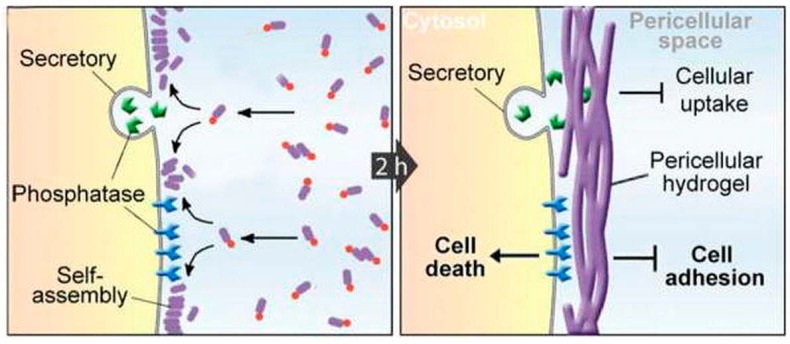
Enzyme catalyzed formation pericellular hydrogel/nanonets to induce cell death. Adapted with permission from ref. [75], Copyright © 2014 WILEY-VCH Verlag GmbH & Co. KGaA.

Novel therapeutic paradigms are being developed that incorporate the unique properties of self-assembling short peptides. In one interesting example, the selective formation of a hydrogel “nanonet” in the pericellular space around cancer cells was demonstrated possible through the use of precursors that assemble only when processed by dephosphorylase enzymes overexpressed by cancer cells (Figure 4). In this way, the nanonet effectively blocks cellular exchange selectively for cancer cells, eventually leading to apoptosis [75]. As another example, combination of self-assembling Phe-Phe, polydopamine spheres, and Fe_3_O_4_ magnetic nanoparticles blended different properties into novel nanostructures that formed adhesive nanotubes and magnetic hydrogels under mild conditions, creating scope for stimuli-responsive biomaterial use, for instance for drug delivery or tissue engineering [76].

## 7. Other Applications

A number of other applications are emerging for Phe-Phe-based self-assemblies. For instance, coupling of the fluorinated dipeptide with l-DOPA yields adhesive coatings that resist protein adsorption and prevent biofilm formation, thus showing potential for their use on a variety of substrates for health-care or marine sectors [16]. The remarkable stability of Phe-Phe nanotubes gives them potential for integration into nanoelectromechanical and medical devices [77]. In the field of biomolecule detection, immobilization of peptide nanotubes onto electrode surfaces may assist in the sensitive detection of ethanol or glucose by amperometric biosensors [78].

Another original study showed the very unique use of the Phe-Phe as a fuel for supramolecular motors. Phe-Phe can be incorporated into the well-ordered nanosized pores of a metal-organic framework (MOF) structure. Upon release, the dipeptides self-organize into a hydrophobic domain that lowers the surface tension of MOF on the released side, resulting in motion of the MOF with the as-generated surface tension gradient via Marangoni effect. This work opens the way to the use of reconfigurable molecular self-assembly based on MOFs and short peptides towards smart autonomous motors for the biomimicry of bacteria swimming motion and/or for the harvest of target chemicals through the integration of recognition units [79].

Recently, a number of interesting properties have been shown for Phe-Phe self-assembled nanostructures, including photoinduced ferroelectricity [80] and piezoelectricity [81], giving scope for functional nanomaterials that respond to electrical or mechanical stimuli. Non-linear optical effect of a second harmonic generation has also been reported, with efficient frequency conversion from NIR to green and blue light, as well as an effect of nonlinear optical waveguiding [82]. Besides, an earlier work showed that photoluminescent peptide nanotubes can arise from the incorporation of lanthanide complexes during the self-assembly process [83]. Further development and use of these properties could realize the next generation of electrophotonic devices.

## 8. Conclusions

We have shown that Phe-Phe is a powerful and useful minimalist building block to drive molecular self-assembly into a variety of nanostructures, as well as hydrogels. These compounds can be produced by a variety of methods, making them easily accessible to scientists coming from different backgrounds, thus allowing rapid progress in their study and application. Besides their innovative use in cancer therapy, biomaterials science, and drug delivery, remarkable properties are continuously being identified giving new horizons for further development of Phe-Phe-based self-assembling compounds towards the next generation of nanomedicine solutions.
